# Magneto-Optical Relaxation Measurements of Functionalized Nanoparticles as a Novel Biosensor

**DOI:** 10.3390/s90604022

**Published:** 2009-05-26

**Authors:** Konstanze Aurich, Gunnar Glöckl, Stefan Nagel, Werner Weitschies

**Affiliations:** University of Greifswald, Institute of Pharmacy, F.-L.-Jahn-Strasse 17, 17487 Greifswald, Germany; E-Mails: gunnar.gloeckl@uni-greifswald.de (G.G.); stefan.nagel@uni-greifswald.de (S.N.); werner.weitschies@uni-greifswald.de (W.W.)

**Keywords:** magnetic nanoparticles, magneto-optical relaxation, immunoassay, IGF-1 assay

## Abstract

Measurements of magneto-optical relaxation signals of magnetic nanoparticles functionalized with biomolecules are a novel biosensing tool. Upon transmission of a laser beam through a nanoparticle suspension in a pulsed magnetic field, the properties of the laser beam change. This can be detected by optical methods. Biomolecular binding events leading to aggregation of nanoparticles are ascertainable by calculating the relaxation time and from this, the hydrodynamic diameters of the involved particles from the optical signal. Interaction between insulin-like growth factor 1 (IGF-1) and its antibody was utilized for demonstration of the measurement setup applicability as an immunoassay. Furthermore, a formerly developed kinetic model was utilized in order to determine kinetic parameters of the interaction. Beside utilization of the method as an immunoassay it can be applied for the characterization of diverse magnetic nanoparticles regarding their size and size distribution.

## 1. Introduction

Since the first utilization of gold nanoparticles in an immunoassay for human chorionic gonadotrophin in the form of a pregnancy test in 1980 [1], nanomaterials are an inherent part of immunological methods. Currently, nanomaterials include quantum dots and metallic nanoparticles as improved labels as well as optical reporters. Nanowires as label-free biosensors and superparamagnetic nanoparticles are used for magnetic separation of biomolecules. The latter were applied in heterogeneous (e.g. enzyme linked immunosorbent assays – ELISAs) [2,3] or homogeneous immunoassays, where signals are detected magnetically by superconducting quantum interference devices (SQUIDs) [4,5], fluxgate sensors [6] or susceptibility measurements [7].

Biosensor systems based on biomolecular recognition are the most widely used analytical technology in biodiagnostics, including the determination of antigens, hormones and drugs by means of antibody application [8]. Antibodies offer quality characteristics, which predestine them for the application in immunoassays: the selectivity to bind to an extremely high variety of molecules, cells or viruses, the high binding specificity and the high bond strength between antibody and antigen. Since nanotechnology found its way into bioanalytical methods, analyses on a minimized scale are possible, which allows for simultaneous detection of numerous analytes and reduced sample volumes.

This contribution concentrates on a homogeneous immunoassay of insulin like growth factor 1 (IGF-1) and its polyclonal antibody anti-IGF-1 with magnetic nanoparticles (MNPs) as signal generators. IGF-1 (7.7 kDa) is the most important peripheral mediator of growth hormone action [9] and it is mainly synthesized in the liver in response to growth hormone stimulation [10]. It has been found that the risk of cancer, diabetes and acromegaly is higher among people with raised blood levels of IGF-1 [11,12]. Thus, determination of IGF-1 levels for diagnosis of these diseases and monitoring during therapy is of crucial importance.

For this purpose iron oxide core-shell-nanoparticles were functionalized with antibodies. The appropriate antigen was added in different amounts. An increase in particle diameter as a consequence of nanoparticle aggregation due to the antigen-antibody-interaction was observed by the measurement of the relaxation time of MNPs before aligned in an external magnetic field. Particle relaxation generates a change in the polarization state of a laser beam, which is propagated through the ferrofluid. This optical signal is detected by a photodiode. The measurement setup allows the simple and fast determination of biomolecular binding events due to the explicit relaxation time detection of only magnetic particles. Interaction analyses are possible in any media and body fluids. Time consuming washing steps are not required [13].

In addition, kinetic parameters such as the interaction rate constants and the equilibrium constant *K_D_* of the underlying protein interactions can be calculated in comprehension with an ad hoc developed kinetic model [14]. In this model we assume a chain like aggregation of MNPs due to antigen-antibody reaction. From the known antigen concentration added to the magnetic antibody sensors and the particle sizes increasing during protein interaction we are able to calculate the unknown parameters *K_D_* and the antibody amount bound on MNPs by means of a scaled plot. However, in principle any biomolecular binding system can be analyzed by the described method.

Beside the application of the method as a homogeneous immunoassay, it can be utilized for the characterization of diverse MNPs concerning their mean particle size and size distribution without laborious sample preparation.

## 2. Experimental Section

### 2.1. Magnetic Nanoparticles

For the immunometric assay described herein DDM128N nanoparticles (Meito Sangyo, Japan) were selected. They are composed of a maghemite core and a carboxydextran shell. In addition to mean size and size distribution measurements by measurements of the magneto-optical relaxation of ferrofluids (MORFF) and dynamic light scattering measurements (photon correlation spectroscopy, PCS), particles were characterized by PCS measurements with respect to their stability, as determined by the zeta potential in diverse media [water, phosphate buffer 10 mM pH 7.4, phosphate buffered saline (PBS) and human plasma].

Since MNPs possess a wide size distribution they were separated in different size fractions by magnetic fractionation. This was done by means of an adjustable electromagnet (Bruker, Germany) and MACS LS columns (Miltenyi Biotec, Germany). For the next preparation steps only MNPs of the largest fraction with a mean hydrodynamic diameter of about 55 nm were utilized.

Functionalization of the particles was achieved by reductive amination. As functionalization agent streptavidin (IBA GmbH, Germany) was applied as it forms extremely stable complexes with biotin. Subsequently one of the interaction partners can be biotinylated and than easily connected with the streptavidinated MNPs [13,14]. Furthermore, direct coupling of protein interaction partners on MNPs by the periodate method was successful as well. Here, biotinylated polyclonal anti-IGF-1 antibody (US Biologicals, USA) was conjugated with the manufactured streptavidin-MNPs. For this purpose, 50 μg of the antibody were diluted in 6 mL PBS under sterile working conditions followed by the dropwise addition of 500 μL of the streptavidin-MNPs. After incubation for 2 h at 4 °C, 100 μg biotin were added in order to saturate the remaining streptavidin binding sites. After another hour, MNPs were washed via a MACS LS column in a static magnetic field.

### 2.2. Magneto-optical Relaxation Measurements of Ferrofluids

#### 2.2.1. Measurement Setup

**Figure 1.**
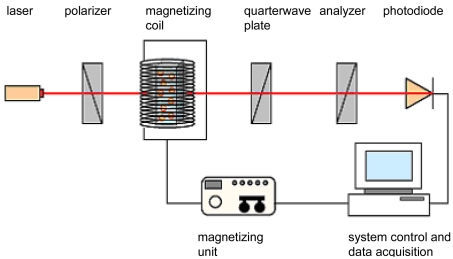
Measurement setup of MORFF.

Suspensions of magnetic nanoparticles are superparamagnetic. That means that they do not offer remanence without an external magnetic field. In the presence of an external magnetic field the particles align along the field direction and relax due to Brownian motion after removing the field. Magneto-optical relaxation measurements are performed by means of magnetic incitation of MNPs and subsequent detection of the relaxation time by a generated optical signal. For this purpose a laser (wavelength 635 nm), a polarizer, a magnetizing coil, a quarterwave plate, an analyzer and a photodiode as the detector are arranged on an optical bench (Figure 1).

Inside the magnetizing coil the cuvette with suspended MNPs is placed. As ferrofluids become anisotropic in the presence of a magnetic field birefringence of the impinged laser beam is generated (Cotton-Mouton-effect) [15]. After switching off the magnetic field the birefringence relaxes similarly to the MNPs due to Brownian motion. The decay of the birefringence is detected as decreasing light intensity by the photo diode. The signal is converted into a voltage by a low noise current amplifier. Assuming monodisperse particles the decay of the birefringence can be described as a light intensity *I(t)* by:

(1) $$I(t)=I_{0}\exp\left[-t/\tau_{B}\right]$$

with *τ_B_* being the Brownian relaxation time.

First of all, particle sizes are determinable with this setup as the relaxation time *τ_B_* is mainly dependent on the hydrodynamic particle diameter *d_hyd_* according to the Brownian equation:

(2) $$\tau_{B}=\frac{\pi \eta {d_{\text{hyd}}}^{3}}{6kT}$$

with *η* being the viscosity of the medium and *kT* the thermal energy. Particle sizes determined by MORFF are in good accordance with sizes generated by dynamic light scattering measurements and plausible when compared with atomic force microscopy (AFM) [16].

Besides mean hydrodynamic diameters the size distribution *P(d_hyd_)* of particle systems can be determined. The superposition of signals from MNPs with different relaxation times results in:

(3) $$I(t)=\int I_{0}(d_{\text{hyd}})\exp[-t/\tau (d_{\text{hyd}})]\cdot P(d_{\text{hyd}})\text{d}d_{\text{hyd}}.$$

**Figure 2.**
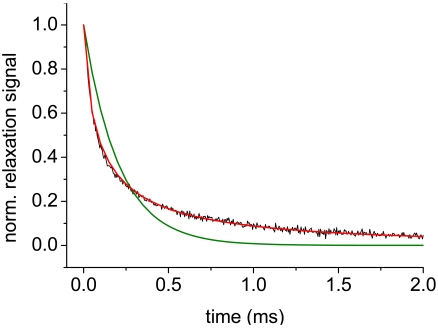
Normalized relaxation data of DDM128N particles of 55 nm in diameter (black) fitted with monoexponential decay (green) and size distribution function (red).

Applying a log-normal distribution function:

(4) $$P\left(d\right)=\frac{1}{\sigma \sqrt{2\pi }}\frac{1}{d}\exp\left[-\frac{{\left(\ln d-\mu \right)}^{2}}{2\sigma^{2}}\right]$$

parameters μ and σ, describing the diameter of the distribution center and the distribution width, can be calculated. Fitting the experimental relaxation curve with the size distribution function (Equations 3 and 4) results in a better conformance of experimental and fitting curve, which shows that the particle diameters in the ferrofluid are size distributed MNPs and not monodisperse (Figure 2).

#### 2.2.2. Monitoring Antibody-Antigen Interactions

In addition, this method can be utilized for the detection of biomolecular interactions concerning quality and kinetic aspects in the manner of a homogeneous immunoassay. A suspension of anti-IGF-1 sensors with an iron content of about 0.1 μM were diluted 10-fold in phosphate buffer 10 mM, pH 7.4, PBS or human plasma (10%) to a final volume of 1 mL in a glass cuvette. Different amounts of IGF-1 antigen were added. Data were acquired every minute for 2 h. In order to exclude unspecific binding reactions, an addition of 3 nM bovine serum albumin instead of antigen served as a control.

### 2.3. Kinetic Model for Immunometric Analyses with MORFF

The binding affinity between antigen (A) and antibody (B) is usually described with association and dissociation rate constants *k_a_* and *k_d_*, or with the equilibrium constant *K_D_*. These quantities are defined by:

(5) $$K_{D}=\frac{k_{d}}{k_{a}}=\frac{c_{A}c_{B}}{c_{AB}},$$

where *c_A_* and *c_B_* denotes the concentration of unbound interaction partners and *c_AB_* the bound ones. These concentrations cannot be measured directly in our experimental setup; instead the binding reaction can be characterized by the rate and end value of particle growth caused by the aggregation of MNPs. In order to calculate the kinetic parameters a model of chain-like aggregation of the particles was developed. Antibody loaded MNPs are linked via the antigen molecules to chains of the form …-[A-A]-[B-B]-[A-A]-[B-B]-…. Two antibodies B on the same MNP are symbolized by [B-B], whereas [A-A] denotes the antigen molecule with two binding sites (epitopes) A. From the known antigen amount added to antibody sensors and the increase in particle size during the interaction the unknown parameters can be estimated using theoretical concepts of stepwise polymerization. A more detailed description of our model is given in [14].

Relating the normalized mean particle diameter in steady state (90 – 120 min incubation time) $D^{SS}={\bar{d}}^{SS}/d_{1}^{SS}$ (d^SS^_1_ – diameter of a single MNP) to the weight-average of chain length distribution the equation:

(6) $$\sqrt{c_{A,0}}\cdot \frac{2D^{SS}}{\sqrt{{\left(D^{SS}\right)}^{2}-1}}=\frac{c_{B,0}+K_{D}}{\sqrt{c_{B,0}}}+\frac{1}{\sqrt{c_{B,0}}}\cdot c_{A,0}$$

with *c_A,0_* being the epitope concentration and *c_B,0_* the antibody binding sites on MNP at the reaction start can be obtained.

For the scaled plot suggested here, the mean diameters *d̄^SS^*, determined by the exponential fit (Equation 1) of the relaxation signals of various antigen concentrations under steady state conditions, have to be normalized by the initial value *d^SS^_1_* to obtain *D^SS^*. Plotting the quantity:

(7) $$y=\sqrt{c_{A,0}}\cdot \frac{2D^{SS}}{\sqrt{{\left(D^{SS}\right)}^{2}-1}}$$

against the respective antigen concentrations *c_A,0_* a linear regression in form of *y* = *ax* + *b* can be performed.

The equilibrium constant *K_D_* and the antibody concentration *c_B,0_* result from the regression parameters as:

(8) $$c_{B,0}=\frac{1}{b^{2}},\;K_{D}=\frac{ab-1}{b^{2}}.$$

## 3. Results and Discussion

### 3.1. Characterization of DDM128N

**Figure 3.**
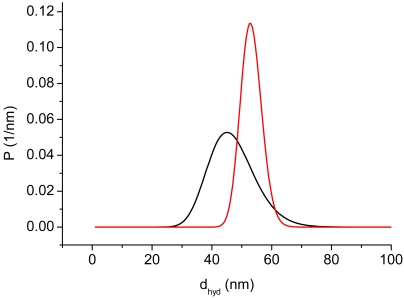
Size distributions of the original DDM128N (black) and the largest fraction (red).

The mean hydrodynamic diameter of the original ferrofluid is about 45 nm. For the application in immunological analyses DDM128N was fractionated into five fractions by magnetic fractionation. Since only the largest particles give a sufficient relaxation signal, the particles of this fraction were used. In Figure 3 size distributions of the original and the largest fraction of DDM128N are depicted. The mean hydrodynamic diameter of the last is about 55 nm.

The zeta potential as a stability marker generated by PCS in different media averaged out at -35 mV in water, -29 mV in phosphate buffer pH 7.4 10 mM, -27 mV in 0.9% NaCl solution, -0.4 mV in PBS and -2.3 mV in 10% human plasma. Stability of the particle suspension decreases with increasing ion and protein concentrations due to the electrostatic interference of repulsive forces between MNPs.

### 3.2. IGF-1/anti-IGF-1 Binding Assay

**Figure 4.**
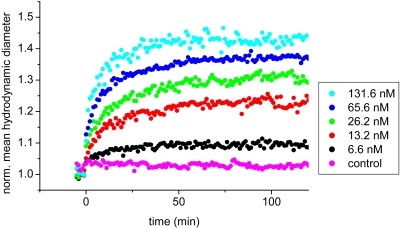
Normalized mean hydrodynamic diameter determined by monoexponential fit of relaxation data of the interaction between anti-IGF-1-MNPs and IGF-1 in different amounts. Control: 3 nM BSA.

In Figure 4 the increase in the hydrodynamic diameter as a consequence of the addition of antigen to antibody sensors is depicted. The hydrodynamic diameters of aggregates developing during the interaction were determined by the monoexponential fit (Equation 1). Over a period of 120 min concentrations of 6.6 nM and higher IGF-1 generated an aggregate size growth up to 1.5-fold of the original particle size. As the diameter increases fast during the first 30 min, a slower part follows. Control experiments include the incubation of antibody sensors with BSA in order to exclude unspecific binding. In this case, MNP diameters remained constant. Steady state conditions are achieved within 90 – 120 min.

### 3.3. Kinetic Aspects of the IGF-1/Anti-IGF-1 Interaction

Among the qualitative determination of the IGF-1/anti-IGF-1 binding reaction in form of the binding assays the kinetic aspects received priority. The knowledge of interaction rates and affinities facilitate the identification and characterization of antibodies and other proteins, e.g. in high throughput screening methods in search of new drugs, where affinity methods are applied [20].

The equilibrium constant *K_D_* was determined by creating a scaled plot. In contrast to the usual immunoassay techniques, where the fractions of bound and free reaction partners can be presented in a linearized plot (e.g. Scatchard plot), these quantities are not directly ascertainable with this method.

The known parameters total antigen concentration and particle size increase due to the formation of aggregates are included in the data evaluation. The model acts on the assumption of the formation of a chain like topology of particle aggregates. Up to trimers the involved MNPs have no alternative for aggregate formation. With the formation of tetramers the possibility of branching is given. That means that the developed model is only valid for small reaction conversions.

**Figure 5.**
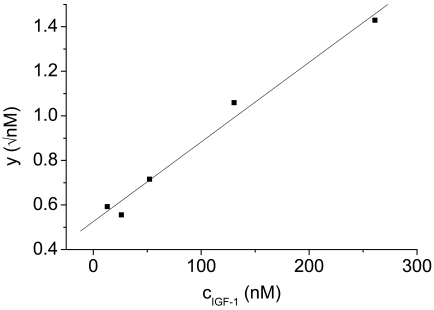
Scaled plot for the interaction between IGF-1 and the antibody sensors.

In Figure 5 the scaled plot for the interaction between IGF-1 and its antibody is depicted as the conversion parameter y versus the concentration of IGF-1 epitopes. The conversion parameter is composed of the underlying steady state diameters averaged from diameters at t = 90 - 120 min and the epitope concentration. The equilibrium constant *K_D_* was calculated as described in Section 2.3. and totals 70 nM. The antibody loading on MNPs *c_B,0_* was estimated as 78 nM.

The formation of MNP chains is already described in literature. Chantrell *et al.* [21] demonstrated chain formation under influence of an external magnetic field. However, also without magnetic fields chain like aggregates due to magneto-statical interactions are described by Klokkenburg *et al.* [22]. Although protein binding plays the important role in aggregate formation in this contribution magnetic dipole-dipole interaction cannot be excluded.

A further prerequisite for the model validity is that only two antibody binding sites on each MNP are involved in the binding process as well as two epitopes per antigen. Former investigations concerning the determination of effective binding sites on functionalized MNPs result in the calculation of 1 – 10 binding sites assuming a particle core diameter of 10 nm [14]. Thus, the assumption of two binding sites is in the right size range.

Modelling of the aggregate formation plays also an important role. We assume an additive increase in diameters, i.e. dimers possess the twofold diameter of monomers, etc. Today we are not able to substantiate the actual aggregate topology. For a further clarification of the aggregate relaxation behaviour additional investigations are required enabling a more concise description of the aggregate behaviour, e.g. size exclusion chromatography ore magnetic fractionation.

The equilibrium constant describing the affinity between antigen and antibody was also determined by other workgroups with different immunometric methods. Manes *et al.* [23] calculated a *K_D_* of 0.1 – 100 nM for IGF-1 and monoclonal antibodies on the basis of BIAcore™ SPR interaction analyses. Another group determined equilibrium constants of 0.01 – 0.15 nM for the interaction between monoclonal anti-IGF-1 antibodies and an IGF-1-analogue (LR3-IGF-1) by ELISA [24]. SPR analysis and also ELISA are methods based on interactions on solid surfaces. First of all MORFF is representing a solution based method, though immobilization of antibodies on MNPs constrains the free movement of proteins. At least a comparison with results from SPR analysis is possible. Day and colleagues [25] obtained comparable kinetic data from SPR, isothermal titration calorimetry and stopped flow fluorescence, whereas the last two methods are solution based. Furthermore, the dextran surface of the SPR sensor chip is not a rigid and solid system, but rather a swollen gel matrix, which enables some degree of diffusional rotation of proteins. Binding constants determined by SPR are very sensitive of potential artefacts produced by mass transport, non-specific binding and avidity effects [26], which require a very accurate and time-consuming sample preparation. The topology of interactions should be present previous to the experiments in order to evaluate the data correctly with evaluation procedures provided by the BIAcore™ software.

Due to the utilization of a polyclonal antibody it is supposable that the *K_D_* determined by MORFF differs from that in literature. A polyclonal antibody is a blend of diverse antibody subtypes with different affinities. We used a polyclonal antibody in our method for demonstration purposes. For detection of kinetics with immunoassays, i.e. using antibodies, concrete data for *K_D_* are rarely; in fact affinity ranges are given (e.g. in [23]). This is due to the numerous method evaluation factors, which cannot be directly influenced. Therefore, the estimation of the order of magnitude of *K_D_* is satisfying.

### 3.4. Method Validation

For a prospective utilization in an every day lab routine the method described herein was validated according the ICH tripartite guidelines [27] for MNPs as well as for the analyzed protein system. Concerning the particle concentration the MORFF technology offers a limit of detection (LOD) of 100 fmol·mL^-1^ particles [14]. Meanwhile other methods offer higher sensitivity regarding particle detection; however, our method is more robust and does not require magnetic shielding [28, 29]. Furthermore the LOD of the analyzed antigen IGF-1 was determined. Ideally, it should be below the physiological and pathological concentrations. In table 1 concentrations of human IGF-1 and the LOD are demonstrated.

The results demonstrate that at present we are able to determine adequate physiological and pathological IGF-1 concentrations with the method described herein. IGF-1 is detected by other workgroups mainly by radioimmunoassays, fluorescence immunoassays, chemiluminescence immunoassays or ELISAs. The LOD of these immunoassays is between 0.1 and 100 ng/mL [34,35]. A major problem of these immunoassays is the interference of IGF-1 with IGF-1 binding proteins (IGFBP), which also appear in plasma samples. Beside elimination of IGF-1/IGFBP-complexes with acidification processes or acid-ethanol extraction, the addition of IGF-2 is now the method of choice to circumvent this problem. IGF-2 binds to IGFBP to the same degree as IGF-1 and blocks binding sites on IGFBP for IGF-1, which then can be analyzed undisturbed with high specific antibodies [12]. Of course this is of major relevance for the sample preparation of the magneto-optical immunoassay described herein. In future experiments, an adequate IGF-2 amount as a sample adjuvant has to be evaluated in order to specify the exact IGF-1 concentration.

In principle the LOD of both, MNPs and protein systems can be decreased by optimization of the cuvette geometry concerning the sample volume. A volume of 10 μL would be enough to ensure the propagation of the laser beam. Furthermore, the application of more appropriate MNPs offering high shape anisotropy, a narrow size distribution and sufficiently large magnetic cores would result in higher signals.

## 4. Conclusions

Measurements of the magneto-optical relaxation of magnetically functionalized antibodies present a novel, simple and inexpensive diagnosis tool for the analysis of biomolecular interactions. The method can be applied as a homogeneous suspension immunoassay, which can be performed in everyday laboratory routines due to its small size and time-saving analysis procedure. Beside interaction analyses kinetic parameters for affinity analyses can be calculated by means of a kinetic model. Furthermore, the method can be applied for the simple and rapid characterization of diverse magnetic nanoparticles regarding their hydrodynamic size and size distribution.

## Figures and Tables

**Table 1.** Physiological and pathological concentrations of IGF-1 in humans (examples) and current LOD of MORFF.

**Physiological concentration**	**Pathological concentration**	**LOD of MORFF**
Children (before puberty): 130 – 485 ng/mL [30]	100 – 150 μg/mL Type 2 diabetes [32]	24.2 ng/mL
Adults: 95 – 250 ng/mL [31]	1 – 3 μg/mL traumatic brain injuries [33]	
